# Hallucinated citations produced by generative artificial intelligence may constitute research misconduct when citations function as data in scholarly papers

**DOI:** 10.1080/08989621.2026.2645390

**Published:** 2026-03-15

**Authors:** David B. Resnik, Mohammad Hosseini

**Affiliations:** a National Institute of Environmental Health Sciences, National Institutes of Health, Durham, NC, USA;; b Department of Preventive Medicine, Northwestern University Feinberg School of Medicine, Chicago, IL, USA;; c Galter Health Sciences Library and Learning Center, Northwestern University Feinberg School of Medicine, Chicago, IL, USA

**Keywords:** Hallucinated citations, research misconduct, fabrication, publication ethics, generative artificial intelligence

## Abstract

In this article, we discuss the growing problem of hallucinated citations produced by Generative Artificial Intelligence (GenAI) in scholarly research and writing. We argue that GenAI hallucinated citations might qualify as a provable instance of research misconduct under the U.S. federal regulations when a) the researcher uses a GenAI tool to produce hallucinated (i.e., nonexistent) citations for a research document; b) the citations function as data because they directly support research findings, as in, for example, review articles or bibliometric studies; and c) the researcher demonstrates indifference to the risk of fabrication of the data (i.e. citations) because they did not check the GenAI’s output for veracity and accuracy. Other types of problematic citations such as bibliometrically incorrect citations, or contextually inaccurate citations, are indicative of poor scholarship and irresponsible behavior, but do not qualify as research misconduct. Recognizing that GenAI hallucinated citations could be regarded as research misconduct in certain cases will hopefully encourage researchers to take this problem more seriously than they do now. In partnership with scientific institutions, funders and professional societies, the scholarly community should work on establishing, promoting, and enforcing standards for responsible use of AI in research, including standards pertaining to citation practices.

## The problem of GenAI hallucinated citations

A well-known problem with Generative Artificial Intelligence (GenAI) tools powered by large language models (LLMs) is their propensity to produce “hallucinated” citations (Nishisako, Higashi, and Wakao 2025). While industry reports have shown that the hallucination rate for some GenAI tools, such as OpenAI’s ChatGPT, has dropped due to the implementation of validation processes (see OpenAI 2025), hallucinated citations are still a cause for concern. Although we are confident that most scholars and scientists understand the importance of good citation practices, we believe that some are not taking the problem of GenAI hallucinated citations seriously enough, and may not realize that in certain situations, including a hallucinated citation in a manuscript may constitute provable research misconduct (at least under U.S. federal regulations).

The problem of GenAI hallucinated citations is unlikely to go away completely because it is inextricably linked to how LLMs operate (Asai et al. 2026; Jones 2025; Kalai et al. 2025; Open AI 2025). LLMs are trained on trillions of words which are converted into tokens. Each token is mapped to a vector in a high-dimensional embedding space developed during the training. Words that are likely to occur together in natural language are also closer to each other in the embedding space. For example, “dog” is close to “puppy” in an LLM embedding but further away from “dinosaur.” During training, models learn how to predict sequences of words. When these predictions miss their mark (that is, when human users, do not consider them to be correct), we call them hallucinations. Since current GenAI tools do not provide estimates of the degree of certainty concerning their responses, it can be difficult for end-users to know whether or when a model is hallucinating. Currently, the only way to make this determination is to compare GenAI answers to ground truths, such as checking whether a proffered citation exists.^1^

When the hallucination problem first became widely known, some GenAI users regarded it as ‘just an annoyance.’ The fact that a computer program could produce anything like a coherent essay, story, or literary analysis was so miraculous that many were willing to overlook this flaw. However, as GenAI tools transitioned from being a source of entertainment to a widely used tool, the hallucination problem has become much more consequential. For example, attorneys have been fined by judges and even dismissed from law firms for submitting legal briefs containing GenAI hallucinated citations (Langham 2024; Legal 2025).^2^ In response to these cases, the American Bar Association issued a formal opinion on responsible use of GenAI tools in legal practice (2024).^3^

GenAI hallucinated citations can also pose serious problems for scholarly publication. A hallucinated citation may support flawed hypotheses or theories, lead researchers down blind alleys, waste scarce resources and undermine the overall integrity, reliability, and trustworthiness of research. In scholarly publications, there have been some noteworthy examples of hallucinated citations. For instance, in a paper published in the journal *Academic Ethics*, 19 out of 29 citations were hallucinated (Retraction Watch 2025). In another study, retracted by *PLOS ONE*, 18 out of 76 cited citations were hallucinated (PLOS ONE Editors 2024). In a perspective piece published in *Digestive Diseases and Sciences* (Springer Nature), 12 of the 14 cited references were hallucinated (Retraction Watch 2026). A study of citations generated by GPT4o (Omni) in the field of mental health (*n* = 176) found that 56% of citations contained errors, with one in five being hallucinated, although the hallucination rate among specific disorders varied between 6% and 46% (Linardon et al. 2025). These are but a few examples that have been identified publicly and made it to the scientific blogs and news websites. Since numerous researchers are now using GenAI to write content, it is conceivable that many publications include GenAI-generated citations, including hallucinated ones.

Additionally, government funding agencies are now having to deal with the problem of hallucinated citations in grant proposals. In the summer of 2025, grant review administrators working for the National Institute of Health (NIH) were inundated with hundreds of proposals written with the help of GenAI tools (Quinn 2025). Some of these proposals may contain hallucinated citations. In response to unethical uses of GenAI in grant applications, the NIH has indicated that if improper AI use is detected in a grant after it is awarded, the matter may be referred to the Office of Research Integrity (ORI) to determine whether research misconduct has occurred (NIH 2025).

## Defining research misconduct

Including GenAI hallucinated citations in a manuscript is poor scholarship and ethically irresponsible, but does it constitute research misconduct? To the best of our knowledge, no publication ethics or research integrity expert has stated publicly that it does. We used to hold that view. However, we have changed our minds and now believe that in some cases, hallucinated citations might qualify as research misconduct. To clarify our position, we will distinguish between ethical and legal senses of “research misconduct” (Resnik 2003).

From an ethical perspective, research misconduct is misbehavior that is widely recognized by the scientific community to be highly unethical. Research behaviors form a continuum from those that are highly ethical (e.g., honest reporting of all relevant data and results) to those that are highly unethical (e.g., data fabrication or falsification), with questionable research practices (e.g., p-hacking or authorship misattributions) falling somewhere in the middle (Shamoo and Resnik 2022; Bouter 2024). Although thinking of research misconduct from an ethical perspective is useful for promoting integrity in science and teaching responsible conduct of research courses, here we are concerned with the legal sense of this term. From a legal perspective, research misconduct is misbehavior that is defined in statues, regulations or institutional policies. Legally speaking, research misconduct does not form a continuum, and a given act either is or is not misconduct.

Here we will focus on the U.S.’ misconduct regulations because they have a significant influence over how misconduct is conceptualized and addressed around the world.^4^ In the year 2000, after more than a decade of deliberation and debate, the U.S. Office of Science and Technology Policy (OSTP) settled upon this definition of research misconduct:

Research misconduct is defined as fabrication, falsification, or plagiarism in proposing, performing, or reviewing research, or in reporting research results. Fabrication is making up data or results and recording or reporting them. Falsification is manipulating research materials, equipment, or processes, or changing or omitting data or results such that the research is not accurately represented in the research record. Plagiarism is the appropriation of another person’s ideas, processes, results, or words without giving appropriate credit. Research misconduct does not include honest error or differences of opinion.

(OSTP 2000: 76262)

This policy (which is followed by most U.S. federal agencies that fund research) defines three types of misbehavior as misconduct: fabrication, falsification, and plagiarism (FFP).^5^ Although some legal definitions of misconduct used by U.S. universities or other organizations include misbehaviors other than FFP (e.g., serious violations of human or animal research regulations), almost all definitions include, at minimum, FFP (Resnik 2019; Resnik et al. 2015; Resnik, Peddada, and Brunson 2009).^6^

Besides defining research misconduct, OSTP also stipulated three requirements for *proving* an allegation of research misconduct. To do this, an institution must find:

1. There be a significant departure from accepted practices of the relevant research community; and
2. The misconduct be committed intentionally, or knowingly, or recklessly; and
3. The allegation be proven by a preponderance of evidence (OSTP 2000: 76,262).^7^

## Research misconduct involving GenAI use

Some cases involving unethical use of GenAI tools would clearly qualify as provable research misconduct under U.S. federal policies, for example:

- **Fabrication/falsification**: A researcher uses a GenAI tool to create fake data or images included in a manuscript submitted for publication or grant application (Resnik et al. 2025).
- **Plagiarism**: A researcher uses a GenAI tool to write a manuscript or grant application. GenAI generates text identical to substantial portions of a previously published article, and the original source is not cited (Resnik and Hosseini 2025).

Cases such as these are not likely to be controversial because most scientists, ethicists, and attorneys would agree that these misbehaviors fall within the scope of the definition of research misconduct. A key issue in these situations will be whether the accused party had the requisite *mens rea*, that is, whether they acted intentionally, knowingly, or recklessly. When evidence indicates that the respondent committed FFP, and did so intentionally or knowingly, the proof of misconduct is fairly straightforward. Proving misconduct based only on recklessness can be more difficult though.

Indeed, making a finding of research misconduct based on the defendant’s reckless behavior (without it also being intentional or knowing)^8^ has only happened in a few cases (Caron et al. 2025; Resnik et al. 2017).^9^ In 2024, the U.S. Public Health Service, which funds NIH-supported research, defined recklessness as:

To act recklessly means to propose, perform, or review research, or report research results, with indifference to a known risk of fabrication, falsification, or plagiarism

(Public Health Service 2024 at 93.234)

Other agencies have used similar definitions of recklessness. The key idea here is that a person’s *mental state* crosses the line from carelessness to recklessness when they demonstrate indifference to the harmful outcomes (such as FFP) of their behavior (Caron et al. 2025). A reckless researcher, like a reckless driver, does not care about the dangers they pose to others.

Applying the recklessness definition to the FFP cases mentioned above, we would argue that a researcher could be liable for provable research misconduct if they use a GenAI tool recklessly. We believe that, given GenAI’s known propensity to hallucinate facts and citations, and to plagiarize text (see Ananya 2025; Nishisako, Higashi, and Wakao 2025; Wiggers 2024), if a researcher uses a GenAI tool without checking its output for possible FFP, then they are acting recklessly because they have shown indifference to the risk of FFP. Responsible use of GenAI tools requires human oversight, which entails, at the bare minimum, checking their output for veracity, accuracy, and validity (Resnik and Hosseini 2025).

## Could GenAI hallucinated citations qualify as provable research misconduct?

Now that we have described some situations in which irresponsible use of GenAI tools could constitute provable research misconduct, we can turn to the case of hallucinated citations. Before explaining why we propose some types of hallucinated citations be considered as research misconduct, providing a definition of hallucinated citations and distinguishing these from two other types of problematic GenAI citations is necessary (see Table 1). It should be noted that this is a non-exhaustive list of problematic citations.^10^ Moreover, all these problematic citations could be due to human or GenAI error, although we hypothesize that hallucinated citations generated by human researchers are quite rare. While humans can also make up citations, we are not aware of any articles or news stories reporting such occurrences.

Putting this all together, would a hallucinated citation ever qualify as FFP? One of these three possibilities can be eliminated quickly: A hallucinated citation does not qualify as plagiarism because it is not misappropriation of another person’s work. A hallucinated citation is giving credit, but it is doing so falsely, because the cited work does not exist.^12^ What about fabrication or falsification? At first blush, a hallucinated citation would seem to not qualify as either of these two types of misconduct, because a citation is not data *per se*. A hallucinated citation is not like a fabricated or falsified lab notebook or spreadsheet entry, or a duplicated or manipulated image. But could citations *sometimes* qualify as data? Although the U.S. Federal research misconduct regulations do not define research data, the Office of Management and Budget, has defined it as:

The recorded factual material commonly accepted in the scientific community as necessary to validate research findings.

(Office of Management and Budget 1999)

This is similar to a widely used definition from the European Parliament and the Council of 20 June 2019 on open data and the re-use of public sector information:

“[R]esearch data” means documents. .. other than scientific publications, which are collected or produced in the course of scientific research activities and are used as evidence in the research process, or are commonly accepted in the research community as necessary to validate research findings and results.

(EU 2019, Article 2, 9)

According to both of these definitions, a citation would count as data when it is *necessary to validate research findings*. In most cases, citations, by themselves, are not necessary to validate research findings. In most research documents, citations serve purposes other than validating research findings, such as giving credit to authors who have helped shape the published literature, situating the work within a certain domain, providing background information for the research, justifying the methods used in the study, or substantiating data interpretations. However, we believe there are at least two types of cases in which citations are necessary for validating the findings of the research: review articles and bibliometric research.

In review articles, the research aims to describe, analyze, or interpret the published literature to reach a particular finding (or findings). The methodology involves identifying, classifying, and curating various references and extracting qualitative or quantitative data from them (Amobonye et al. 2024; Ghosh and Choudhury 2025). In bibliometric research, the purpose is to discover patterns and trends in the published literature. Bibliometrics research involves identifying, classifying, mapping, and analyzing various references (Donthu et al. 2021). In both types of research, hallucinated citations would directly affect scientific findings and could qualify as data fabrication.

For example, let’s consider a hypothetical systematic review of the literature on the adverse drug reactions related to a particular medication. If the review finds that a particular reaction is mentioned in fiveout of 100 articles, but none of these five cited articles exist because they are GenAI hallucinations, we could say that the researcher fabricated data because the cited articles are necessary to validate the study findings. Or, let’s consider a hypothetical bibliometric study that attempts to measure the annual growth of the average number of authors per article in a certain discipline. If some of the articles used in this study do not exist because they are GenAI hallucinated citations, we would say that the researcher fabricated the data because the cited articles are necessary to validate the study findings.

Of course, a responsible researcher writing a review article or performing a bibliometric study is not likely to include hallucinated citations because they will be drawing their citations from reliable sources, such as scholarly indexes and journal websites. However, suppose that a researcher uses a GenAI tool to help write text and produce citations for a review article or bibliometric study without checking its output for veracity, accuracy, and validity. If the resulting article includes GenAI hallucinated citations, the researcher would be liable for provable research misconduct because they fabricated data (i.e., citation) and they did so recklessly by demonstrating indifference to the risk of data fabrication. This is reckless because GenAI tools have a well-known tendency to produce hallucinated citations, but the researcher did not even bother to check the tool’s output for validity.

Finally, while we think that a hallucinated GenAI citation could qualify as data fabrication when the citation functions as data, we do not think it would qualify as data falsification because the GenAI tool is making up a citation, not changing an existing citation.

## Objections and replies

Objection: Treating hallucinated citations as a form of data fabrication is stretching the definition of research misconduct in the U.S. federal regulations beyond what it was intended to cover.

We recognize that considering hallucinated citations as a form of data fabrication is a novel (and perhaps even radical) idea that may not square with preconceived ideas about what it means to fabricate data. When scientists and policymakers crafted the U.S. federal government’s definition of research misconduct over two decades ago (Resnik 2003, 2019), they probably were not thinking about GenAI hallucinated citations. However, the scientific practices frequently change in response to new technologies (Hosseini et al. 2022). The use of GenAI has led to numerous challenges to science’s ethical norms that, such as questions about whether AI systems should be named as authors on papers or whether they should assist with peer review (Resnik and Hosseini 2025). At this juncture, it is important to raise questions as to whether hallucinated citations should ever qualify as research misconduct to help the research community conceive of ways to address this problem and promote ethical use of GenAI. Accordingly, we believe that our suggestion is not stretching the definition but applies the proposed rationale in the existing regulation to a new (and evolving) situation.

Objection: Since the scientific literature may now contain hundreds of GenAI hallucinated citations, treating these cases as possible research misconduct may overwhelm academic institutions with misconduct allegations.

We recognize that our view creates potential enforcement concerns for academic institutions, but we think these problems are not as dire as they may seem. First, we are not proposing that academic institutions *should* retrospectively audit the scientific literature to identify hallucinated citations and bring misconduct charges against perpetrators. Institutions must decide how to respond to our arguments and conclusions. Going forward, we hope that researchers and institutions become more aware of the seriousness of GenAI hallucinated citations and take appropriate steps to prevent it. Second, we are not claiming that every hallucinated citation qualifies as research misconduct. We are only claiming that hallucinated citations might qualify as provable research misconduct when citations serve as data to support research findings. In cases where hallucinated citations do not serve as data, they are still ethically questionable and should be corrected (if possible), but they do not rise to the level of misconduct.

We believe that it should be up to the research institutions to devise appropriate sanctions for researchers who include GenAI-generated hallucinated citations in research documents. In supporting this view, we believe that publishers and journals should invest in automated tools that detect hallucinated citations, and that they should report them to researchers’ institution, if appropriate. Potential penalties should be proportionate to the nature, degree and frequency of a researchers’ offense. We anticipate that by recognizing that hallucinated citations might qualify as research misconduct in certain cases, the demand for citation accuracy would increase and researchers would be encouraged to take hallucinated citations much more seriously than they do now. Moving forward, we urge institutions to establish, promote and enforce standards about responsible use of AI in various research disciplines, including standards pertaining to citation practices.

## Concluding discussion: The future of problematic citations

Regardless of whether hallucinated citations produced by GenAI tools qualify as misconduct, they undermine the truthfulness, reliability, and integrity of research. This is a significant problem that needs more attention. Even before the advent of GenAI tools, the scientific community was struggling to prevent or weed out problematic citations (Hosseini et al. 2020). For example, contextually inaccurate citations come in various forms and range from subtle confusions to serious and perhaps even malicious misrepresentations. According to a recent meta-analysis (Baethge and Jergas 2025), almost 17% of all quotations in medical publications have inaccuracies, and roughly half of those inaccuracies involve major errors (i.e., “the claim is not at all supported by the source”). This rate of inaccuracy could certainly be compounded and exacerbated by GenAI limitations mentioned in the introduction. Perhaps novel AI tools could help us locate and report these problematic citations, but then again, we would have to deal with numerous publications that need to be corrected or retracted – a logistical nightmare. This may explain why some journals have been discovered to engage in the unethical practice of *stealth corrections*, that is, “post-publications changes, without providing any indication that the publication was temporarily or permanently altered” (Aquarius et al. 2025). If publishers are, or will be engaged in more stealth corrections, the community would not be able to track and hold reckless researchers accountable. Instead correcting articles in secrecy, publishers should do so openly and transparently, in coordination with research institutions. In any case, we believe that citation ethics deserves more attention within the research integrity and publication ethics literature, and welcome additional conceptual and empirical research in this domain, including critiques of the views presented in this editorial.

## Figures and Tables

**Table 1.** Types of problematic GenAI citations.

Problematic citation	Definition	Example	Notes
Bibliometrically incorrect citation^11^	The citation refers to a real scholarly or creative work, but it includes some minor bibliometric errors, such as a misspelled name, incorrect publication year, or DOI.	According to Kuhn, scientific progress does not always occur through the steady accumulation of knowledge but is often marked by revolutions in which existing paradigms are overthrown. Kuhn, Tomas. 1963. *The Structure of Scientific Revolutions*. Chicago, IL: University of Chicago Press.	Although Thomas Kuhn has authored *The Structure of Scientific Revolutions*, and stated the presented claim, this citation contains two bibliometric errors: the correct publication year is 1962, not 1963, and “Thomas” is misspelled.
Contextually Inaccurate citation	The citation refers to a real scholarly or creative work, but the citing author has misinterpreted the work or removed it out of context.	According to Kuhn (1962), adherence to ethical standards is important for scientific progress. Kuhn, Thomas. 1962. *The Structure of Scientific Revolutions*. Chicago, IL: University of Chicago Press.	This citation misinterprets what Kuhn said in *The Structure of Scientific Revolutions*. Kuhn did not make any claims about scientific ethics, in the sense of professional moral duties or normative rules governing scientific behavior.
Hallucinated citation	The citation refers to a creative or scholarly work that does not exist.	According to Kuhn, ethical standards for research are important for promoting the public’s trust in science. Kuhn, Thomas. 1998. *The Ethics of Science and Technology in the 20*^*th*^ *Century*. Chicago, IL: University of Chicago Press.	This is a hallucinated citation because Kuhn never wrote this book, which should be obvious to a competent scholar who studies the history, philosophy, and ethics of science. However, the citation looks real, especially to someone who does not know the literature well.

## Footnotes

1. Since LLMs generate their output by predicting word sequences, there is a sense in which every GenAI citation is hallucinated because it has been made up! However, people usually do not refer to these word predictions as hallucinated when they happen to exactly match existing citations.

2. In one of the first known occurrences of GenAI hallucinated citations in legal practice, Peter LoDuca, an attorney representing Roberto Mata in personal injury lawsuit against Avianca Airlines, used six hallucinated GenAI citations in a legal brief he submitted in response to Avianca’s motion to dismiss the case. The problem was discovered when an attorney for the defendant told Judge Kevin Castel he could not locate the citations in any legal databases. Judge Castel conducted his own search for the citations but could not find them either. Eventually, LoDuca told Judge Castel that he had assigned Steven Schwartz to do the legal research for the brief and that Schwartz had used ChatGPT to help write the document. Outraged by this unprofessional conduct, Judge Castel fined the attorneys $5,000 (Mata v Avianca, Inc, 2023).

3. For more instances of hallucinated citations in law, see Damien Charlotin’s (2026) database. Of the 946 listed cases on February 16, 2026, 647 were hallucinated, while 192 misrepresented facts or precedent, and 107 had false quotes.

4. Most of the misconduct definitions used in other countries are similar to the U.S. definition (Resnik, Rasmussen, and Kissling 2015). Additionally, when a U.S. agency, such as the NIH, funds a research project involving a collaboration between investigators based in the U.S. and in other places, the agency’s misconduct regulations would apply to the project. Also worth noting is that the U.S. is the largest funder of research and development (R&D) in the world, and that roughly 18% of U.S. R&D is federally funded (National Science Foundation 2025b).

5. It is important to note that the National Science Foundation addresses the use of AI tools in its newly revised definition. The NSF states that research misconduct may be committed by an individual directly or “individual directly or through the use or assistance of other persons, entities, or tools, including artificial intelligence (AI)-based tools” (National Science Foundation 2025a).

6. The definition of research misconduct in the European Code of Conduct for Research Integrity defines research misconduct as “fabrication, falsification, or plagiarism. .. in proposing, performing, or reviewing research, or in reporting research results (All European Academies 2023, 10).”

7. To have “preponderance of evidence” for a statement means that given the evidence, it is more likely than not that the statement is true.

8. Almost all of ORI’s published findings of research misconduct find that the defendant acted “intentionally, knowingly, or recklessly” without distinguishing between these different concepts (Long et al. 2025). This is not a very helpful practice, because it obfuscates important facts about the case, such as the evidence concerning the defendant’s state of mind.

9. For example, in 2015, the National Science Foundation (NSF) found that Frank Sauer recklessly committed data fabrication and falsification for splicing, reusing, labeling or manipulating digital image data. Sauer admitted that the data had been fabricated or falsified but he said this had been done by someone else – a rival trying to get him in trouble. A federal judge reviewing Sauer’s appeal of the case said that publishing fabricated and falsified images in this fashion amounted to reckless behavior because it showed indifference to the truth (Resnik et al. 2017).

10. For example, there are citation cartels, coerced citations, excessive self-citation, and so on (Bruton et al. 2025; Plevris 2025). In this paper, we are not concerned with every type of ethically problematic citation but only GenAI hallucinated citations.

11. We recognize that a bibliometrically incorrect citation could be so far off the mark that one could consider it to be hallucinated, but discussing the difference between a wildly incorrect citation and a hallucinated citation is outside the scope of this piece.

12. Because a hallucinated citation makes a false claim, it could be a form of criminal or civil fraud under federal or state law. Fraud is commonly defined as “A knowing misrepresentation of the truth or concealment of a material fact to induce another to act to his detriment” (Black’s Law Dictionary 1999, 7). Legally, fraud is different from research misconduct, which falls under administrative law (for sanctions administered by government agencies, such as the Office of Research Integrity) or contract law (for sanctions imposed by employers, such as universities). In this article, we focus on research misconduct, not fraud. However, our claims may have implications for fraud as well.
